# “It’s never just one thing”: understanding risk factors for sports injuries in track and field

**DOI:** 10.1186/s13102-026-01719-4

**Published:** 2026-04-29

**Authors:** Kalani Weerasinghe, Ranil Jayawardena, Indu Nanayakkara, Andrew P Hills

**Affiliations:** 1 School of Health Sciences, UTAS Health, University of Tasmania, Launceston, Tasmania Australia; 2 Health and Wellness Unit, Faculty of Medicine, University of Colombo, Colombo, Sri Lanka; 3 Department of Physiology, Faculty of Medicine, University of Colombo, Colombo, Sri Lanka; 4 Department of Physiology, Faculty of Medicine, University of Peradeniya, Kandy, Sri Lanka

**Keywords:** Athletic stakeholders, Injury risk factors, Injury surveillance, Sports injuries, Track and field

## Abstract

**Background:**

Track and field athletes are vulnerable to injury due to numerous intrinsic and extrinsic factors, such as previous injuries, high training loads, inadequate recovery, and psychological stress. However, there is limited information about the lived experiences of athletes from low- and middle-income countries.

**Objective:**

This study aimed to explore perceptions of risk factors for sports injuries among Sri Lankan track and field athletes and key stakeholders.

**Methods:**

This qualitative study employed convenience sampling to recruit elite athletes, their parents, and diverse stakeholders, including doctors, physiotherapists, coaches, sports administrators, and academics. Face-to-face, semi-structured interviews were audio-recorded, transcribed, and analysed using reflexive thematic analysis.

**Results:**

Thirty-two participants completed the study (12 athletes, 17 stakeholders, and 3 parents). Five overarching themes related to injury occurrence and persistence were apparent among participants, including: (i) recurrent injuries due to inadequate recovery and load management; (ii) nutritional deficits, limited recovery, and psychological stress increasing vulnerability; (iii) environmental and equipment limitations elevating biomechanical load and fatigue-related risk; (iv) fragmented injury-management pathways and non-standardised return-to-play decisions; and (v) minimal injury monitoring and documentation, which limited prevention and continuity of care. Lower-limb injuries were most common, often associated with high training loads, poor recovery, technique errors, suboptimal ground conditions, and unsuitable equipment. Stakeholders highlighted nutrition, hydration, recovery strategies, and mental stress as key contributors. Injury management was inconsistent, with many athletes self-managing or relying on coaches rather than multidisciplinary healthcare. Formal surveillance systems were largely absent, prompting stakeholder calls for a coordinated digital record-keeping approach.

**Conclusions:**

Sports injury prevention and management in Sri Lanka are constrained by limited facilities, suboptimal nutrition and recovery practices, and insufficient injury monitoring systems. Addressing these challenges requires integrated, multidisciplinary strategies that strengthen nutrition, recovery, psychological support, and record-keeping systems to optimise athlete health and performance.

**Supplementary Information:**

The online version contains supplementary material available at 10.1186/s13102-026-01719-4.

## Introduction

Sport is more than just a physically demanding activity; it plays a vital role in adolescents’ physical, mental, and social development by fostering discipline, and a healthy lifestyle [1]. Despite the health benefits, participation in sports training and competition is associated with injuries, which can have a substantial impact on both athletes and society [2]. Among sporting disciplines, track and field events are associated with different types of injury, often due to the distinct technical demands and physiological requirements of each discipline [3]. Distance runners are commonly affected by lower limb overuse injuries such as stress fractures [4], while sprinting and jumping events tend to result in a higher incidence of acute injuries [3]. Additionally, evidence suggests that high jumpers and pole-vaulters are more susceptible to traumatic injuries often due collision with the ground, whereas athletes in throwing events frequently experience both overuse and acute injuries, particularly affecting the upper body [5].

Injury incidence in track and field varies considerably across different levels of competition and training intensities and ranges from 1 to 30 injuries per 1,000 athletic exposures (AE), with higher rates consistently reported during competition compared with training [5]. A cross-sectional analysis spanning one Olympic cycle (2012–2016) indicated that 64% of athletes sustained at least one injury, with the majority occurring during high-intensity training or competitive phases for which lower limb injuries accounted for 83% (*N* = 524) of the ten most common injury types [6]. Across 14 international athletic championships (2007–2018) there were 928 injuries among 8,925 male competitors, 597 injuries among 7,614 female competitors [7], with the highest injury proportion of injuries occurring during sprint events in both men (24%) and women (26%) [7]. Sports injuries are increasingly recognised as having significant psychological impact. Injured athletes may experience heightened symptoms of depression and anxiety due to factors such as a loss of identity, reduced social support, fear of re-injury, and prolonged absence from sport [8, 9]. Notably, recent evidence indicated that female athletes are up to three times more likely to develop depressive symptoms following injury compared with male athletes, highlighting sex-related differences in mental health outcomes following injury [10].

Multiple epidemiological studies about athletic injuries have identified a range of risk factors including a history of previous injuries [3], being male [11] or older [3], participation in combined events or middle- to long-distance running [7], high training loads [3], the use of spike shoes [12], psychological factors such as self-blame [13], and a history of lifetime physical or sexual abuse [14]. Despite these findings, the understanding of injury risk factors in athletics in middle to low income countries remains limited, highlighting the need for continued investigation into potential contributors to athletic injuries [15].

Several qualitative studies have explored sports injuries associated with track and field disciplines and includes the report of many athletes competing at the international level who struggled to recognise and interpret symptoms of ill health and injury by themselves [16]. Importantly, as their training loads increased, the need for medical support exceeded what could be provided by parents or local communities. In the absence of a structured transition of healthcare responsibility to sports organizations, athletes were often left to manage their health issues independently. This previous study also reported that reducing training load was the most common approach to treating sports injuries [16]. A qualitative study involving youth athletes aged 12–15 years in Sweden explored the underlying causes of sports injuries and found that injuries were not solely attributed to individual-level factors and were perceived as outcomes of interactions across multiple levels [17]. This previous study highlighted three key contributing factors including inadequate knowledge related to youth athletic development during daily practice, narrow and short-term approaches within coaching communities and sports policies, and broader societal health behaviours [17]. A previous study explored the opportunities and challenges related to injury prevention and management as perceived by coaches, allied health professionals, and general practitioners with the finding of key barriers to performance and prevention being limited education and inadequate resource availability [18]. Further, it was highlighted that was a need for leadership and stakeholder engagement to promote enhanced education and better resourcing in the management of sports injuries [18].

Given the adverse performance, health, and economic consequences associated with injuries in track and field, injury prevention represents a critical priority for athletes and stakeholders [19]. Injury surveillance has been widely recognised as a foundational component of effective injury prevention strategies [20], as it enables systematic identification of injury patterns and associated risk factors [21]. However, the factors which may contribute to injury or prevention of injury for athletes from countries with low to middle with no exploration of the socioeconomic status such as Sri Lanka are not known with information about the lived experiences and perceptions of athletes and key stakeholders regarding the underlying risk factors for sports injuries in track and field within the Sri Lankan context. Therefore, this study aimed to explore how athletes and stakeholders perceive, experience, and describe the risk factors which may contribute to sports injuries in track and field athletes in Sri Lanka.

## Methods

### Design and study population

This qualitative study employed a convenience sampling approach to recruit participants. Track and field athletes registered with the Sri Lankan Athletic Association were recruited across disciplines including sprinting, middle- and long-distance running, throwing, and jumping (Table 1). Athlete performance level was classified using the athlete performance calibre framework described by McKay et al., [21] which provides a standardized taxonomy based on competitive level and training demands. In this study, elite athletes were defined as those competing at international level (e.g., Asian Championships, Commonwealth Games, or equivalent) or representing Sri Lanka at senior international competitions, while highly trained athletes were defined as national-level competitors regularly participating in national championships with structured, high-volume training but without sustained international representation.

Stakeholders from the Institute of Sports Medicine, including medical doctors, nutritionists, sports physiotherapists, sports psychologists, and sports masseurs; sports administrators from the Ministry of Sports; International Olympic Committee-qualified nutritionists; national-level athletics coaches affiliated with the Ministry of Sports; physical training teachers and athletic trainers; and athletes’ parents to provide a diverse range of experiences and perspectives were recruited (Table 2). Parents of participating athletes were purposively included due to their involvement in training decisions, access to healthcare, and injury management, particularly within the Sri Lankan context. Parents were not required to meet the professional or athletic eligibility criteria applied to athletes and stakeholders. Individuals were excluded if they had a current injury persisting for more than six months or a prior history of doping violations.

All participants provided written and informed consent after receiving a detailed explanation of the study aims and procedures. The study was conducted in accordance with the Declaration of Helsinki and received ethical approval from the Institutional Ethical Review Committee, Faculty of Medicine, University of Peradeniya, Sri Lanka (Reference: 2025/EC/61).

### Data collection

Demographic information including age, gender, athletic event (for athletes/parents) or professional role (for stakeholders), and years of experience was collected at the beginning of each interview. One-to-one in-depth semi-structured interviews were conducted face-to-face by the principal investigator (initials here) using an interviewer-administered guide developed in line with the study objectives.

Separate interview guides were developed for (i) athletes and their parents, and (ii) stakeholders. To determine the experience and perspective of athletes and their parents, interview questions were focused on four topics including (1) injury history and perceived causes, (2) physical factors related to sports injuries, (3) nutrition, recovery, and psychological factors influencing injury risk, and (4) environmental and sports equipment-related risk factors (Supplementary File 1). For stakeholders, the four topics were: (1) physical factors related to sports injuries, (2) nutrition, recovery, and psychological factors influencing injury risk, (3) environmental and equipment-related risk factors, and (4) injury monitoring and record-keeping practices (Supplementary File 2).

Open-ended questions and probing techniques were used to explore participants’ perceptions and lived experiences in detail. While the interviews began with structured questions aligned to each topic, the interview was flexible, allowing participants to elaborate freely. Each interview lasted for up to 30 min and conducted in a private and comfortable setting to encourage open discussion. The principal investigator maintained a neutral stance, providing guidance, ensuring focus, and stimulating constructive dialogue without leading participants. Handwritten notes were recorded during the interviews, and non-verbal cues were observed where relevant. With prior consent, all sessions were audio-recorded using a digital voice recorder (Olympus WS-853, Olympus Corporation, Japan), a reliable and widely used device for qualitative research, to ensure accurate data capture and transcription.

### Data analysis and rigor

Data were analysed using reflexive thematic analysis [22, 23], underpinned by an interpretivist/constructivist epistemology that views knowledge as co-constructed between participants and researchers. An inductive, primarily semantic, approach was adopted, while also attending to latent meanings when participants’ narratives reflected underlying cultural or systemic issues [24]. The analytic process followed Braun and Clarke’s six phases: (i) familiarisation with the data through repeated reading of transcripts and listening to audio recordings; (ii) generating initial codes systematically across the dataset in NVivo v10.0; (iii) searching for themes by collating codes into potential patterns; (iv) reviewing themes against coded extracts and the entire dataset; (v) defining and naming themes through iterative refinement and discussion; and (vi) producing the report by integrating analytic narratives with illustrative participant quotations. Trustworthiness of the data was enhanced through multiple strategies. The principal investigator (KW) maintained a reflexive journal throughout the process to acknowledge positionality and potential influence on interpretation. Reflexivity was further supported by team discussions to critically reflect on assumptions. Dependability was supported by maintaining an audit trail of coding and analytic decisions using NVivo. Confirmability was strengthened through team consensus during theme development and independent verification of the accuracy of Sinhalese-to-English translations by a bilingual researcher experienced in qualitative research. This study was designed, conducted, and reported in accordance with the Standards for Reporting Qualitative Research (SRQR) [25] (Supplementary Material 3) and the Consolidated Criteria for Reporting Qualitative Research (COREQ) guidelines [26] to ensure methodological transparency and rigor (Supplementary File 4).

## Results

**Fig. 1:**
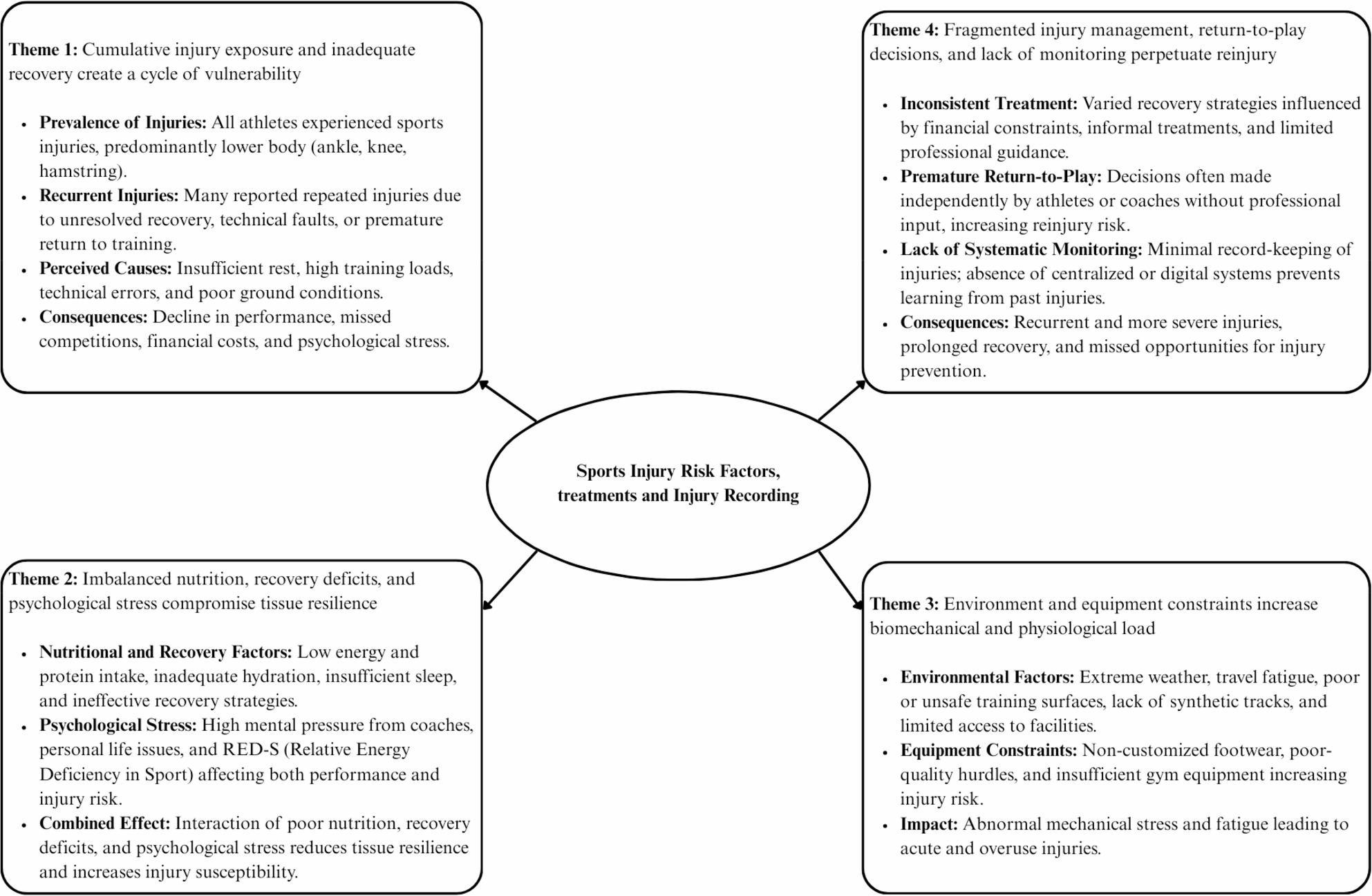
Summary flow of themes emerging from the data

Thirty-two respondents participated in the study, including 12 track and field athletes, 17 athletic stakeholders, and 3 parents. Participant characteristics are summarised in Tables 1 and 2. Five main themes emerged from the semi-structured interviews: (i) cumulative injury exposure and inadequate recovery creating a cycle of vulnerability; (ii) imbalanced nutrition, recovery deficits, and psychological stress compromising tissue resilience; (iii) environmental and equipment constraints increasing biomechanical and physiological load; (iv) fragmented injury management and return-to-play decision-making perpetuating reinjury; and (v) the absence of systematic monitoring preventing learning from past injuries (Fig. 1). These themes reflect interacting biological, behavioural, environmental, and system-level mechanisms influencing injury occurrence and recurrence (Fig. 1).

**Table 1:** Socio-demographic data of athletes (*n* = 12)

Pseudonym	Sex	Age range	Event	Performance level*	Years of competitive experience	Highest education level
Mark	M	20s	Sprinting	Elite	9	School level II
Daniel	M	20s	Sprinting	Elite	11	School level II
Adam	M	30s	Sprinting	Highly trained	12	Diploma
Jack	M	20s	Sprinting	Elite	8	School level II
Peter	M	20s	Middle-distance	Elite	10	School level II
Simond	M	30s	Middle-distance	Highly trained	14	Degree
Walker	M	20s	Long-distance	Elite	9	School level II
Wyatt	M	30s	Long-distance	Highly trained	15	Diploma
Sue	F	20s	Long-Jump	Elite	7	Degree
Susan	F	20s	High jump	Elite	8	School level II
Heily	F	20s	Hammer throwin	Highly trained	9	School level I
Suhaan	M	30s	Javelin throwing	Elite	13	Diploma

Performance level classified using the athlete performance calibre framework described by McKay et al.

(Reference: McKay AK, Stellingwerff T, Smith ES, Martin DT, Mujika I, Goosey-Tolfrey VL, et al. Defining training and performance caliber: a participant classification framework. International journal of sports physiology and performance. 2021;17(2):317 − 31.) [21]

**Table 2:** Socio-demographic data of athletic stakeholders (*n* = 17) and athletes’ parents (*n* = 3)

Pseudonym	Role	Sex	Age range	Years of professional experience^*^	Education level
Dr. Perera	Sports medicine physician	M	40s	18	Postgraduate
Dr. Silva	Sports medicine physician	M	50s	22	Postgraduate
Anzar	Strength and conditioning coach	M	40s	15	Diploma
Soysa	Strength and conditioning coach	M	40s	17	Degree
Romy	Strength and conditioning coach	M	50s	20	Degree
Rivi	Strength and conditioning coach	M	40s	16	Diploma
Tishka	Strength and conditioning coach	M	30s	12	Degree
Averon	Sports physical therapist	M	40s	18	Degree
Lynnara	Sports physical therapist	F	30s	11	Degree
Melissa	Dietitian	F	30s	10	Degree
Dr. Fernando	Sports nutrition physician	M	40s	19	Postgraduate
Roshan	Sports psychologist	M	30s	9	Degree
Shaun	Athletic trainer	M	40s	14	Diploma
Paul	Sports masseur	M	40s	16	Diploma
Prof. Noah	Academic (sport sciences)	M	40s	25	Doctorate
Kyle	Physical training teacher	M	40s	17	Diploma
Shane	Ministry of Sports administrator	M	50s	21	Degree
Harshani	Athlete’s parent	F	40s	-	School level II
Joshua	Athlete’s parent	M	50s	-	School level II
Jane	Athlete’s parent	F	50s	-	School level II

^*^Service experience was calculated only for professional stakeholders; parents were excluded from this variable

### Theme 1: Cumulative injury exposure and inadequate recovery create a cycle of vulnerability

Athletes reported repeated exposure to injuries, which, combined with insufficient recovery, technical faults, and premature return to training, created an accumulating vulnerability to future injuries.

#### Sub-theme I: recurrent injury experiences

All athletes (12/12) reported experiencing at least one sports injury during their careers, most commonly affecting the lower limb (ankle and knee). While participants described multiple injuries, these accounts reflect their experiences rather than serving as direct evidence of unresolved risk. Several athletes described experiencing the same injury multiple times. For example, an elite sprinter claimed that, *“I’ve had a hamstring injury three times. I feel like I’m getting the same injury again and again.”* (Mark).

A parent also reflected on the repeated injuries their child had endured: *“My son has had ankle problems more than once*,* and every time he gets back to training*,* I’m worried it might happen again. It feels like the injuries keep following him.”* (Joshua) These reports highlight that repeated injuries were rarely isolated events. Athletes and parents expressed concern about injuries recurring and the challenges associated with returning safely to training.

#### Sub-theme II: perceived causes of injuries

Participants reflected that repeated injuries often followed from insufficient rest, inadequate recovery, or technical and load-management challenges. These factors were perceived as contributing to vulnerability to subsequent injuries. For example, a sprinter explained, “*I think the immediate reason was that after the race we didn’t have a place to stay that night*,* so my friend and I walked from the ground to Kottawa on foot*,* and I didn’t get a chance to rest.”* (Adam).

Another sprinter highlighted a similar issue, emphasizing the role of training load and coaching practices stating that, *“I feel that it was more to do with the coach. He doesn’t allow us to rest properly; he is killing us with vigorous workouts. Even when we’re given a normal session*,* we’re asked to do heavy loads*,* followed by short bursts. Our muscles get tight*,* and when we suddenly have to do a speed session*,* the muscle just snaps. Actually*,* I even changed my coach because of it. Because of that problem*,* even now I find it hard to do a speed workout. Most of the time*,* when I do a speed workout*,* I have to use tape or some kind of support.”* (Daniel).

A few athletes attributed recurrent injuries to insufficient muscle strength or to technical errors with highly trained sprinter reporting that, *“The reason for my back injury was reduced strength in my back muscles. The ankle injury happened because of an inaccurate technique when I was clearing a hurdle; it hit my leg*,* and one of the tendons in my ankle got torn.”* (Adam).

Poor ground conditions emerged a perceived factor which increased the risk of injury as reported by two long-distance runners. One of the long-distance runners reported that, *“The main reason is the ground condition. Especially when we have to keep running marathons on hard ground every day*,* our muscles get tight*,* and the next day we can easily get injured. At the Bogambara ground I’ve experienced this*,* as it has a lot of uneven patches with water puddles. When three athletes sprint together*,* sometimes one-foot lands higher and the other in a hole*,* and that can cause injuries. Also*,* the ground is overgrown with weeds*,* it’s hard to run because you lose balance. I ran there and ended up with this calf injury.”* (Walker).

#### Sub-theme III: Consequences associated with injuries

Athletes reported catastrophic consequences following their sports injuries, with the primary concern being a decline in performance due to missed training, which often resulted in missing competitions. One athlete explained, *“Honestly*,* my training and performance really dropped after my first calf injury. At the start*,* I was in the national pool*,* but I got removed after the injury. In the second trial*,* I couldn’t run properly*,* and that’s how I was dropped. Because of the injury*,* I also missed the 2019 South Asian meet.”* (Walker).

Another sprinter highlighted the mental impact of injury, describing persistent fear of reinjury and diminished performance and reported that, *“Mentally*,* I’ve had a big setback because now I can’t run even a single race at my top performance. No matter how much I train*,* I just can’t reach my maximum. I also keep worrying in my head that if I try to sprint hard again*,* the injury might come back right there. Honestly*,* I’m not running as well as I used to because of this problem. After the previous injury*,* I had to stop training for about three weeks.”* (Mark).

Athletes also reported substantial financial burdens associated with treatment, which exacerbated psychological stress and created a vicious cycle of missed training and further performance loss. One long-distance runner reported, *“I spent a lot*,* especially when I had a bone fracture earlier*,* and even this time it took about another six months to recover. Getting back to proper training took even longer. During that time*,* because of COVID*,* we had to order medicines through couriers*,* and I mainly got treatment from an Ayurvedic hospital. It was really hard traveling back and forth*,* and usually*,* I had to go about three times a week*,* and the medicines cost around 5*,*000–6*,*000 rupees a day. Being in the Army*,* it was tough not being able to run*,* and when they forced me to*,* I was really affected mentally.”* (Walker).

Parents also expressed concern about these consequences, particularly the disruption to education and family finances with one parent reporting, *“As parents*,* we end up spending so much money and time for treatment*,* sometimes traveling long distances. It affects the whole family. My daughter also misses school because of injuries*,* which makes it even harder for her.”* (Jane).

These accounts highlight the impact of having to manage injuries and the perception that inadequate rest, along with persistent technical or strength deficits, contributes to a cumulative injury burden.

### Theme 2: Imbalanced nutrition, recovery deficits, and psychological stress compromise tissue resilience

Participants reported that a number of factors including inadequate energy availability, protein intake, hydration, sleep, and psychological stress reduce the body’s ability to tolerate training loads, recover effectively, and maintain neuromuscular control increased athletes’ injury risk.

#### Sub-theme I: Nutritional and recovery-related factors and sports injuries

Athletes highlighted the importance of nutrition and recovery techniques as key factors in preventing injuries. A sprinter reported that *“Honestly*,* when I was at my lowest weight*,* I felt like I was just flying over the hurdles; it felt so light. I still feel that way. For my BMI to properly match my height*,* my weight should be around 74 kg*,* but I’m still at 60. I need proper nutrition to increase my weight.”* (Mark).

Maintaining proper hydration was considered critical for preventing cramps and muscle injuries with one sprinter reporting, *“If we don’t focus on hydration*,* we will definitely get cramps. Therefore*,* to avoid that*,* I also use recovery drinks other than water.”* (Jack). In addition, he stressed the importance of adequate rest after training. *“Not resting after training also contributes to injuries. After training*,* we usually need 2 to 2½ hours of sleep.”* (Jack).

A middle-distance runner who was also studying at university emphasized the difficulty of finding time to rest after training due to having to attend lectures and reported, *“After a workout*,* the muscles are tired because they have been worked hard. So*,* if I go and sit in a lecture right after training*,* my muscles don’t get enough rest*,* and that results in stiffness and injuries.”* (Peter).

Athletes reported using structured recovery strategies following training to minimize injury risk and enhance performance. One sprinter described his post-training routine: *“As soon as the workout finishes*,* I do a short jog for about ten minutes*,* walking around 100 meters. After that*,* I do stretching and take a meal with both carbohydrates and protein. Then I rest and sleep for about two hours. For 2–3 times a week*,* I do ice baths and massaging; that really helps me to recover.”* (Daniel).

Stakeholders reinforced the perspectives of athletes and provided further insights regarding injury risk, emphasizing nutrition, hydration, and recovery strategies. Stakeholders emphasized the importance of energy balance, protein intake, and micronutrient monitoring. One Sports Nutrition Physician explained, *“From a nutrition perspective*,* if carbohydrate intake in the diet isn’t adequate*,* it can contribute to injuries.”* (Dr. Fernando).

Stakeholders emphasized the importance of adequate protein intake and energy replacement after training and competitions with a dietitian reporting, *“Because of their high energy expenditure*,* an athlete needs to get enough energy*,* nutrients must be properly absorbed*,* and sufficient glucose must reach the body. If they don’t get enough protein*,* their muscle fibres can break down. Specifically*,* athletes need a certain amount of protein based on their training and body weight.”* (Melissa).

A physical training teacher emphasized that inadequate protein intake exacerbates injury risk. *“I don’t think our athletes even eat 40–50 grams of protein per day. Because of that*,* our bodies don’t really recover properly*,* which may increase the risk of injuries.”* (Kyle).

A structured pre- and during training nutrition plan was emphasized with a physician stating that, *“Yes*,* this is very important to reduce injuries. In particular*,* the amount of food and water an athlete consumes before*,* during*,* and after training is critical. If the athlete goes into a workout without proper carbohydrate loading or protein intake*,* they will inevitably experience energy loss. If the training lasts for two to four hours*,* the athlete must have a healthy snack during training*,* containing carbohydrates*,* protein*,* and some fat.”* (Dr. Fernando).

Adequate hydration was repeatedly identified as a critical factor for injury prevention and optimal performance. Stakeholders noted that dehydration could increase the risk of muscle and ligament injuries, with a sports medicine physician claiming that, *“Severe dehydration and electrolyte imbalance causes ligament injuries like ACL injuries*,* and dehydration can worsen performance.”* (Dr. Silva).

A recurring issue which was reported was the inadequate monitoring of athletes, which was perceived to contribute to overtraining, improper recovery, and heightened their susceptibility to injuries. An administrator stated that, *“Also*,* our athletes are not properly managed they compete throughout the year. Due to economic issues*,* many of them run for money without taking rest. Almost all athletes depend on the Sri Lankan Army. Sometimes*,* because of problems in the Army*,* they also get stuck.”* (Shane).

Stakeholders emphasized that various recovery practices, including sleep, allows the body to repair muscle fibres damaged during training, restore energy levels, and normalize elevated body temperature and blood flow. A dietitian stated that, *“after training*,* blood flow is elevated*,* body temperature is higher*,* and energy levels are depleted. During training*,* muscle fibres can be damaged*,* so proper rest is needed to recover from this stress. Stretching and cooldown exercises help prevent muscle tightness from developing into injuries. A good nutritional recovery plan and quality sleep are equally important.”* (Melissa).

Stakeholders also highlighted the importance of structured training schedules to avoid excessive fatigue with a university academic stating that, *“What really needs to be done is to properly schedule the athlete’s training and competitions throughout the year. Without a well-structured schedule*,* if they just play around all year without organized training and competition planning*,* it can have a significant impact on the athlete.”* (Prof. Noah).

#### Sub-theme II: Psychological factors associated with injury risk.

Athletes highlighted psychological factors as being significant contributors to an increased injury risk, emphasizing the influence of stress, mental focus, and personal life on their performance and susceptibility to injuries. One sprinter stated that he had to end his relationship with his partner to maintain his mental focus and freedom and reported, *“That’s something very valuable to me*,* because I can’t usually run a race without mental freedom. In fact*,* I even broke up with my girlfriend because of this problem. Whenever I had a race coming up*,* she knew about it and kept stressing me out. Honestly*,* the only race where I was able to run really well was the one where I had blocked her.”* (Mark).

Parents also reflected on the psychological pressure athletes face, particularly from demanding training environments. One parent reflected, *“Sometimes I feel the coaches push them too hard*,* even when they are clearly exhausted. My son comes home stressed*,* saying he can’t speak up because he fears being dropped. That kind of pressure not only affects his confidence but also makes him more vulnerable to injuries.”* (Harshani).

Stakeholders underlined the critical role of mental wellbeing in injury prevention and sports performance identifying factors such as personal relationships and addiction to social media as key influences on athletes’ psychological state. One strength and conditioning coach stated, *“Mental health really affects injuries and performance. Love affairs negatively affect their mental health. Also*,* addiction to mobile phones is a big issue. They lose their focus because of these distractions.”* (Romy). The importance of mental focus and pre-event relaxation strategies was further highlighted by a sports masseur who stated that, *“A sprinter running the 200 meters. If their mind isn’t fully present at the start of the event or is focused on something else*,* when they accelerate*,* they could injure their hamstring.”* (Paul). A coach elaborated on the advice he typically gives athletes before a competition to reduce anxiety and stress and stated, *“Before a race*,* we advise the athlete to mentally relax and not focus on other competitors. If they wish*,* they can listen to a song or visualize a role model. At that moment*,* it is important not to put pressure on them.”* (Rivi).

A sports psychologist emphasized that mental health is also linked to nutritional factors, particularly the risk of energy deficiency, stating, *“Another aspect often overlooked is mental health*,* particularly RED-S (Relative Energy Deficiency in Sport). If the energy expended during training is not adequately replenished through nutrition*,* athletes can develop an energy deficiency*,* which may significantly affect their bodies.”* (Roshan).

Nutrition, recovery, and psychological stress were not isolated factors, however, interacted to reduce athletes’ physical and mental resilience to training demands.

### Theme 3: Environmental and equipment constraints increase biomechanical and physiological load

Participants reported that poor ground conditions, extreme weather, travel fatigue, inadequate facilities, and non-customised footwear impose abnormal mechanical stress and fatigue, increased the likelihood of sustaining both acute and overuse injuries.

#### Sub-theme I: Environmental risk factors associated with injury risk

Athletes consistently reported that training and competing in extreme weather, and with substandard equipment, were major risk factors. For instance, one sprinter explained, *“If you don’t warm up properly during rainy days*,* it can cause muscle issues. Also*,* during very hot days*,* we get dehydrated easily.”* (Mark).

Athletes also emphasised that the strain of training on hard, dry surfaces, particularly in regions lacking synthetic tracks increased the risk of injury with one sprinter reporting, *“We don’t have a carpet track here in Wayamba Province. So*,* we have to go to Colombo for training when it’s closer to a meet. It actually takes about 2½ to 3 hours of traveling*,* and because of that tiredness*,* if we train on the same day*,* we often get injuries.”* (Daniel).

An experienced javelin thrower highlighted that Sri Lanka lags behind in adopting modern technologies utilized by most developed athletic nations and reported, *“For example*,* if an athlete runs 100 m*,* we do not have the technology to measure the time taken to cover the first 30 m. We also cannot measure the speed or the angle at which a ball is released from the hand. In this regard*,* we are far behind the technological standards of the world.”* (Suhaan).

Stakeholders reported that there were several environmental factors that increased injury risk. A sports physical therapist pointed out the need to consider heat stress and environmental conditions when planning training sessions stating, *“first*,* we need to check the heat index where we plan to practice. If the heat index is around 32 or 33*,* or otherwise uncomfortably high*,* we don’t do practice that day*,* or we reduce the training load.”* (Averon). A strength and conditioning coach explained, *“In our provinces*,* even if we get tracks*,* we often don’t have synthetic ones. Sometimes*,* there isn’t even a proper track for speed workouts. Because of the conditions on the ground*,* many athletes are affected and prone to injuries.”* (Anzar).

#### Sub-theme II: Sports equipment and footwear factors contributing to injury risk

The absence of appropriate equipment and facilities in the training environment was also identified as a major barrier. Athletes noted that poor-quality hurdles, a lack of gym machines, and limited access to recovery facilities increased the risk of injury. One sprinter reported, *“When we train in the gym*,* the AC does not work*,* and it becomes very hot inside. Even at Sugathadasa ground*,* maintenance is poor*,* and the hurdles are of low quality. When we hit our legs on them*,* it causes injuries. In other countries*,* hurdles are made of fibre-like material*,* but in Sri Lanka those facilities are lacking.”* (Mark).

A hammer thrower emphasized the insufficient facilities in his training environment, explaining their impact on injuries reporting, *“Sometimes we can’t do certain exercises because the gym doesn’t have the necessary machines. As a result*,* we often miss out on practicing those exercises. When the required machines are not available*,* we tend to look for alternative ways to do them*,* and that can lead to injuries.” (Heily).*

Another sprinter from an outstation reported experiencing similar difficulties and reported, *“When we train in Matara*,* we don’t have proper weights or hurdles. All those facilities are available only in Colombo. But traveling to Colombo is really difficult. With the fatigue from the journey*,* training can also lead to injuries. On top of that*,* the expenses are very high.”* (Mark).

Sports footwear was frequently identified as a critical determinant of injury prevention and several athletes emphasized the importance of supportive, good-quality shoes and the risks of continuing to train in worn-out spikes and reported, *“Yes*,* actually my first shin bone fracture happened at a time when I didn’t have money to buy good shoes. I was using a slightly heavy pair*,* and the front part felt tight on my toes. After doctors examined me*,* they told me to change my shoes. From that day onward*,* I’ve only used branded shoes*,* even though they cost around 46*,*000 rupees.”* (Walker). Poor footwear was also identified by a physical therapist and an academic as contributing to injuries who stated, “*For athletes with flat feet*,* there should also be a support system in place. We do maintain a separate database to record each athlete’s injuries and generate reports. Ideally*,* each athlete should have shoes customized according to their specific needs. Without that*,* if they train or compete in improper shoes*,* injuries can occur.”* (Prof. Noah).

Coaches emphasized that the limited availability of proper training equipment, particularly hurdles, posed a significant risk. One coach explained, “Sri Lanka, there aren’t training hurdles available; what we mostly have are competition hurdles. Most often, those hurdles topple even with a light touch, and athletes get injured. So, for athletes training in rural areas, the equipment they need is often lacking.” (Romy).

### Theme 4: Fragmented injury management, return-to-play decisions, and lack of monitoring perpetuate reinjury

Participants reported that without consistent medical oversight, evidence-based rehabilitation, or injury surveillance systems, athletes repeatedly return to training without addressing underlying risk factors, leading to recurrent and more severe injuries.

#### Sub-theme I: Inconsistent treatment pathways delay recovery

Treatment strategies for injury management and recovery varied among athletes, and were often influenced by financial constraints, lack of professional guidance, and over-reliance on informal treatment practices. A high jumper reported, *“At first*,* I used Ayurvedic treatments*,* but they didn’t help much. Later*,* since the Ayurvedic treatments weren’t sufficient*,* I consulted a doctor*,* and that cost a lot. "* (Susan).

Many athletes described adopting self-directed strategies or depending on their coaches for treatment decisions, rather than following evidence-based treatments. Some athletes reported resorting to informal management methods, such as massages from coaches, self-directed rest, or alternative medicine treatments. For example, one sprinter explained, *“I asked my coach to massage the injury site*,* but it didn’t improve. Then I rested*,* and only after that it improved.*” (Jack).

Another athlete highlighted financial barriers to accessing medical care, leading him to rely solely on the physiotherapist available at training camps reporting, *“I couldn’t really go to a doctor because it costs a lot. So*,* I only showed it to the physio at the camp*,* and that’s how I managed the situation.”* (Jack).

Several athletes turned to Ayurvedic medicine as their primary form of treatment, citing both accessibility and cost, though many acknowledged its limitations compared to allopathic care. For instance, a long-distance runner reported, *“When I had the major fracture*,* I went to an Ayurvedic doctor in Mawanella for treatment. At first*,* they told me to take some rest and then try jogging 5–10 minutes to see how it felt and gradually increase from there. After the calf injury*,* I didn’t take much medicine like that. Instead*,* I had needling and vibration therapy*,* which helped a bit.”* (Walker).

Sports medicine professionals emphasized the use of standardized treatment protocols, including RICE (Rest, Ice, Compression, Elevation), physiotherapy, and progressive rehabilitation, as essential for safe recovery and return to play. *“Muscle injuries or ligament injuries are treated with ice and other standard treatments. Different protocols are followed depending on the injury*,* but normally it is managed immediately.”* (Dr. Perera).

#### Sub-theme II: Unstandardised decision-making leads to premature return to sport

Athletes’ decision-making about return-to-play also varied considerably. Some made independent judgments about when to stop or resume training, often without professional input with a sprinter reporting that, *“Soon after an injury*,* I completely stop training for 2–3 days. After that*,* I check the pain during movement and do some stretching to see. If I don’t feel any issues*,* then I resume training*,* and I make that decision on my own.”* (Daniel).

In contrast, an elite sprinter reported that decisions regarding their return to training were primarily made by their coaches or physiotherapists, with one sprinter reporting that, *“When I go for training in the morning*,* my coach usually asks whether I can run with the injury or if it’s still painful*,* even during my warm-up. So*,* my coach is the one who decides when I should stop or restart training after an injury.”* (Mark).

Reports from coaches confirmed the athletes’ experiences, with some coaches stating that they decided when an athlete should stop training or competing after sustaining an injury, without seeking advice from medical professionals. As one strength and conditioning coach reported, *“I usually say to stop training and do ice therapy. After that*,* no other activities are done for at least a day. Then the next day I check whether the pain has reduced. If the recovery is happening*,* then normally I start training for them.”* (Anzar).

In contrast, a sports masseur highlighted the importance of multidisciplinary decision-making in determining when an athlete should resume training, cautioning against coaches or athletes making such decisions in isolation and explained that, *“Given our limitations*,* the main thing we can do is release any tight muscles*,* improve joint range of motion*,* and then refer the athlete to physiotherapy. After that*,* the doctor decides when they can resume training.”* (Paul).

A physical therapist and academics emphasized that return-to-play decisions should be gradual and individualized, based on injury severity, functional testing, and international protocols with the physical therapist explaining that, *“We don’t necessarily stop all training; for example*,* we might reduce running intensity to around 40%. When a hamstring injury occurs*,* if it’s a grade 1*,* we usually don’t put the athlete back on the track in the first week. Initially*,* we focus on strengthening the muscles*,* then gradually progress the load. We strictly follow return-to-play protocols…”* (Averon). This perspective was further supported by an academic who discussed the concept of optimal loading and reported that, *“Most of the time*,* we don’t completely stop training. However*,* we rest the injured site and gradually continue training alongside rehabilitation exercises. For example*,* if someone has a knee injury*,* they can still do exercises for other parts of the body without any problem. Usually*,* we try to rehab the injury gradually…”* (Prof. Noah).

One sports medicine physician explained that injury prevention is a cornerstone of injury management, highlighting the importance of both pre-session and off-session protocols and explained that, *“As a doctor*,* all parts of preventive protocols are important. Pre-session: identify potential injuries*,* like hamstring or ankle issues. Off-season: profile players and group them based on risk”* (Dr. Silva).

#### Sub-theme III: Absence of systematic monitoring prevents learning from past injuries

Athletes relied heavily on their coach or physiotherapist to document or track their injuries, as one sprinter described by stating that, *“I don’t have any records of my injuries; I think my physio has them”* (Mark). Similarly, a long-distance runner admitted, *“I don’t usually track or keep records of my injuries; I only tell my coach”* (Walker). Some athletes further highlighted that they often shared details of their injuries informally with peers rather than with professionals, with one sprinter stating, *“Usually*,* I don’t tell my coach about my injuries; mostly I share them with my peers”* (Mark), and another sprinter reporting: *“I keep my own memory of what happened*,* even though I don’t write it down anywhere*” (Daniel).

In contrast some coaches reported that they did document an athlete’s injury history with a strength and conditioning coach reporting that, *“For the best athletes I work with*,* I’ve asked them to personally keep a record of their injuries. I’ve given them a system for how to write it*,* and they record it themselves. Both the athletes and I have a notebook. When I plan their workouts*,* if an athlete gets injured*,* I note it down with a red pen…”* (Romy).

However, another strength and conditioning coach stated that he does not keep records of his athletes’ injuries stating that, *“No*,* we don’t keep records. We are registered with the Ministry*,* and our sports medicine unit is at Kalubowila Hospital. That’s where we are registered and updated.”* (Soysa).

All stakeholders emphasized the value of systematic record-keeping for injury prevention, management, and research. For example, a sports masseur explained that, *“This is very important because it allows us to predict future injuries. When a new injury occurs*,* we can look at the athlete’s previous injuries and the rehabilitation we provided. "* (Paul). This was supported by a dietitian who stated that, *“By recording injuries systematically*,* we can conduct research*,* review past injuries*,* and analyze the treatments provided. "* (Melissa).

All stakeholders proposed transitioning towards digital, app-based, or software-driven systems to overcome current reliance on handwritten notebooks and fragmented documentation. A sports nutrition physician stated that, *“This reporting system is quite good if it’s app based. Ideally*,* it would be even better if there were a way for the athlete to record their own data directly”* (Dr. Fernando).

## Discussion

This study explored the experiences and perceptions of athletes and stakeholders about the risk factors which may contribute to sports injuries in track and field athletes in Sri Lanka. There was broad agreement across both athletes and stakeholders that sports injuries rarely result from a single cause and are influenced by a complex interplay of physical, nutritional, psychological, environmental, and equipment-related factors. The current study identified differences among athletes and among stakeholders about being different treatment approaches when an athlete is injured and the possible factors which influence guide the choice of treatments. Athletes relied on their coach or physiotherapist to document or track their injuries, and it was evident there was a lack of a centralised or structured approach to injury surveillance. Importantly, all stakeholders confirmed the value of systematic record-keeping related to injury which may guide injury prevention and rehabilitation. Further, stakeholders emphasized the need to transition towards the use of digital technologies to not be reliant on handwritten documentation and may be accessible to all members coaches and healthcare providers. The absence of systematic injury record-keeping among athletes suggests a gap in monitoring practices. This lack of formal documentation may limit opportunities to learn from past injuries, inform return-to-play decisions, or implement prevention strategies, consistent with previous reports highlighting the importance of injury surveillance in track and field athletes [27].

Interviewing elite and high performing athletes in Sri Lanka did reveal that sports injuries are common amongst track and field athletes. and Although injury may be an inherent part of competing at an elite level, some participants have reported that competing and training with the possibility of being injured is not necessary to achieve peak performance [28]. In the current study the report of there being multiple factors which may lead to injury was highlighted by all participants. These included commonly reported risk factors such as training overload, inadequate warm-up, insufficient recovery, and compromised nutrition or mental health, which are well documented in the athletics literature from high-income countries [29]. Importantly, psychosocial stressors, including managing personal relationships, academic pressures, and other external distractions, were also perceived to increase injury risk, aligning with previous evidence highlighting the role of psychological and social demands in injury susceptibility [30, 31]. What distinguishes the present findings is not the identification of these factors alone, but how they coexist within a resource-limited sporting environment. Unlike athletes in countries with well-established institutional support systems, participants in this Sri Lankan cohort described limited access to structured recovery services, multidisciplinary medical teams, and systematic monitoring practices. As a result, common risk factors such as overload or psychosocial stress may be less effectively managed, potentially amplifying their impact on injury risk. These findings extend existing knowledge by demonstrating that while injury risk factors appear broadly similar across contexts, their consequences may be greater in low- and middle-income settings due to systemic and environmental constraints affecting athlete support.

Appropriate nutrition, including carefully selected pre-training meals, post-training meals, and hydration strategies, was reported by athletes in Sri Lanka [32] to positively influence performance, enhance recovery, reduce injury risk, and support overall well-being. Consistent with these insights, a sports psychologist in the current study underscored the role of nutrition and injury risk, and was guided by the International Olympic Committee’s consensus statement that chronic under-eating or low energy availability, leading to RED-S, is a significant risk factor for bone stress injuries among athletes [33] and one which affects both male and female athletes. Consistent with previous studies, participants in the current study reported that inadequate recovery, including disrupted sleep, travel fatigue, and poorly managed routines, increased their susceptibility to injury [34]. However, in the Sri Lankan context, these risks may be further exacerbated by limited access to recovery facilities and support systems, highlighting the influence of environmental and systemic factors on athletes’ recovery practices. In a previous study of 958 athletes, those reporting anxiety or depressive symptoms during the preseason had more than twice the odds of sustaining an injury, with 30% reporting anxiety and 22% reporting depressive symptoms [35]. Similarly, athletes who feel pressured to push beyond their limits may experience poor sleep quality, further elevate their risk of injury while simultaneously impair recovery and performance [36]. In the current study, this was evident among athletes who were studying an undergraduate degree and having to balance their sporting commitments with academic study. This information may need greater acknowledgement from coaches and parents to provide appropriate support, possibly reducing the risk related to injury.

The report of poor training environments characterized by inadequate facilities and limited or low‑quality sports equipment, as described by athletes and coaches, reflects a challenge that may influence injury risk in track and field. Although much of the injury epidemiology literature has focused on intrinsic and workload‑related risk factors such as overuse patterns and biomechanical demands in athletics [37, 38], studies have also emphasized the role of environmental and contextual conditions in shaping athletes’ injury exposures. For example, inadequacies in training conditions and equipment have been highlighted as potential contributors to injury risk in athletic populations and broader sport settings [39], underscoring that suboptimal infrastructure and tools can compromise safe movement and technique. This is particularly pertinent in resource‑limited settings, where athletes may train on surfaces or with equipment that do not meet optimal standards, potentially exacerbating the burden of musculoskeletal injuries observed in track and field athletes across diverse contexts [40].

Both athletes and stakeholders in the current study described that injury management decisions were frequently made independently by coaches or athletes, often without formal consultation with medical professionals. This approach contrasts with evidence from elite sport systems, where integrated performance health management models emphasise that optimal recovery and injury prevention require shared decision-making between athletes, coaches, and qualified medical staff [41]. In professional football, poor cooperation and communication between coaching and medical teams have been associated with higher rates of muscle re-injury, increased injury burden, and reduced player availability [42, 43]. Although much of this evidence originates from team sports, the principles of coordinated, multidisciplinary care are highly relevant to track and field, where training loads, competition schedules, and return-to-play decisions similarly require careful medical oversight. In the Sri Lankan context, limited access to multidisciplinary support structures and formal performance health systems may contribute to greater reliance on coaches or athletes to manage injuries independently. This highlights a critical gap between best-practice recommendations and real-world practice in resource-limited settings and underscores the need for clearer role delineation, improved communication, and greater integration of healthcare professionals in athlete management to optimise recovery and reduce re-injury risk.

Globally, best practice recommends a multidisciplinary approach involving physicians, physiotherapists, sports psychologists, nutritionists, and coaches to guide injury management through evidence-based rehabilitation and structured return-to-play protocols [44]. Following evidence-based rehabilitation protocols improves outcomes and return-to-play safety [44]. It is plausible that in a country with a relatively small amount of money which can be used to support the health of athletes, there may be resistance to increases funding for processes and systems that optimise injury management and prevention. This may lead to the behaviours reported by athletes and some coaches, whereby they often provide care without consultation with healthcare providers and without knowledge of appropriate treatment strategies to optimise athlete recovery. These findings support the need for greater investment in a structured and integrated approach that includes enhanced education for different stakeholder groups and a coordinated approach to athlete management.

Previous studies have emphasized that athlete-engaged record-keeping enhances self-awareness and improves adherence to rehabilitation [45], and not a common behavior for athletes interviewed in the current study. The benefits of documentation include more accurate communication between athletes and support staff such that there may be improvements in the monitoring of and the factors which influence recovery from injury [45]. However, in Sri Lanka, relatively low levels of knowledge about health and well-being may result in athletes not being aware of the need to accurate record about their injuries and training regimen. This effect is further amplified if coaches and healthcare providers do not keep records or share this information with the consent of athletes.

Despite these limitations, there was a strong consensus among respondents that systemic record-keeping is essential for understanding injury trends, informing preventive strategies, and guiding rehabilitation. Stakeholders expressed enthusiasm for transitioning to digital, app-based platforms capable of centralizing injury data, enhancing communication among athletes, coaches, and healthcare professionals, and supporting evidence-based decision-making. Prior studies emphasize the value of shifting from manual logs to digital databases accessible to the full multidisciplinary team [46], as these systems improve data completeness, standardization, and real-time monitoring. In Sri Lanka, adopting mobile or online platforms could help overcome geographical and administrative barriers, particularly if designed to be low-cost, user-friendly, and accessible to both coaches and athletes.

A major strength of this study is its real-world relevance. By incorporating perspectives from athletes, parents, and a wide range of athletic stakeholders, including coaches, physiotherapists, nutritionists, physicians, psychologists, and administrators, it provides a holistic view of injury risk factors in track and field. The use of in-depth interviews and reflexive thematic analysis yielded rich, nuanced insights, while triangulation across multiple groups and reflexive journaling enhanced the credibility and trustworthiness of the findings.

Aligned with a recent systematic review [47], the current findings support a biopsychosocial approach to sports injuries for track and field athletes in Sri Lanka. To date, no published studies specifically from South Asian track and field teams have explored these risk factors, which may be related to a greater risk of sustaining injury. It is possible that athletes in resource-limited settings face similar risks to athletes with greater wealth and access to support from coaches and healthcare providers. However, the relative financial and social burden associated with limited support for injury prevention and treatment may be greater in countries with lower socioeconomic status and requires further investigation.

### Clinical, research, and policy implications

**Fig. 2:**
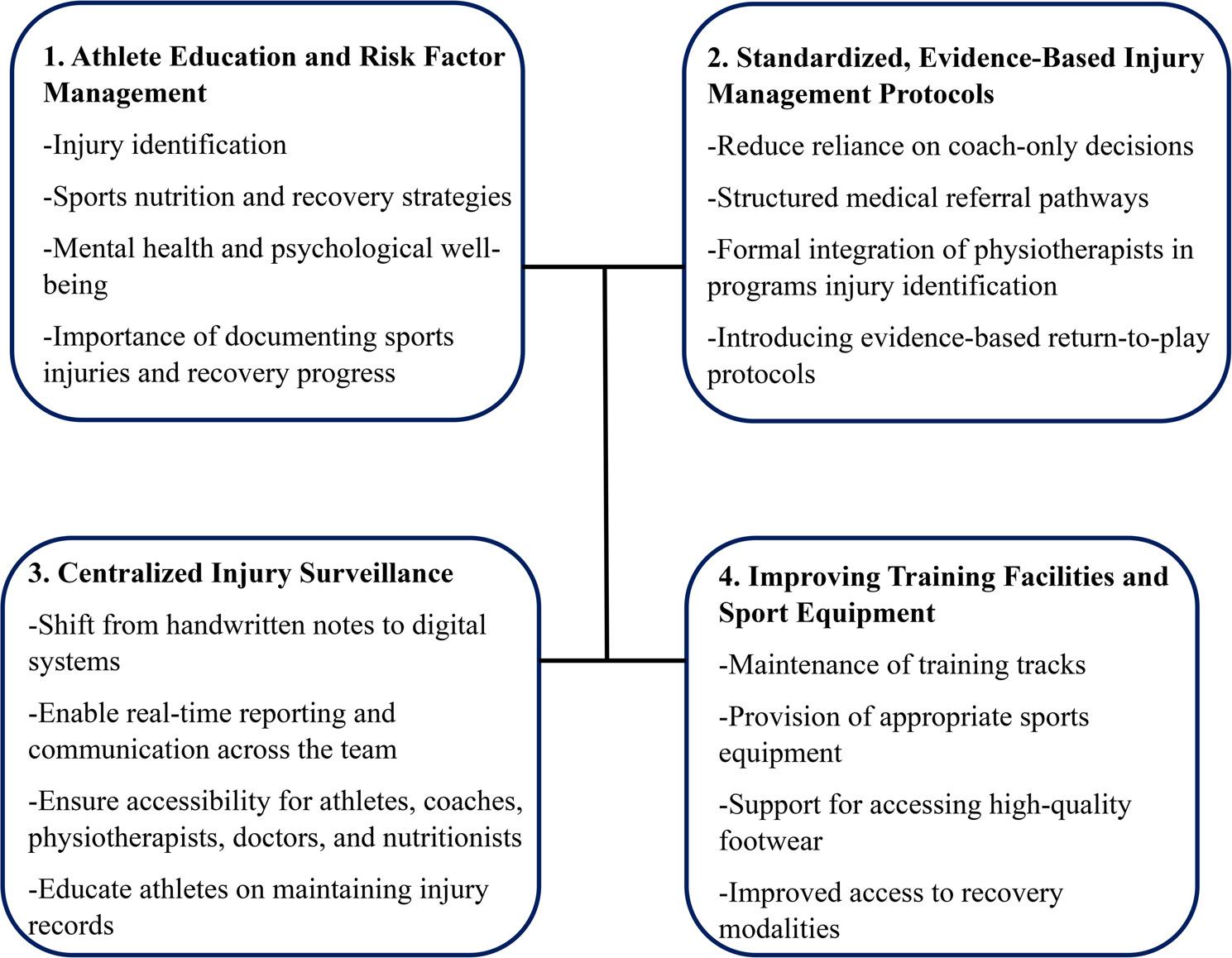
Practical recommendations for injury prevention and management in resource-limited settings

Based on the experiences reported by participants, the findings suggest that a multidisciplinary approach to sports injury prevention and management could be beneficial in Sri Lanka. While many injuries are currently managed without standardized rehabilitation or return-to-play protocols, evidence from sports medicine emphasizes the value of coordinated, multidisciplinary care. Implementing such approaches may also be relevant in other countries with limited resources and support systems for athletes. Currently, athlete support is fragmented, and athletes often have to manage other personal commitments, making it difficult to recover and prevent further injury. There is a strong call to introduce improved systems for injury surveillance within a multidisciplinary environment. Such systems would need to be implemented at a systems and organisational level to be effective. Improved facilities, investment in equipment that minimises injury risk, and greater athlete education on factors that optimise health and recovery, including nutrition, recovery strategies, and psychological health, are needed for both athletes and support staff (Fig. 2).

### Limitations

The use of convenience sampling and a relatively small sample size may restrict the generalisability of the findings, as the sample may not represent the broader population of athletes in Sri Lanka or other countries. Self-reported experiences may introduce bias due to participants’ reliance on recall or social desirability, particularly regarding injury management practices and the use of traditional approaches. Additionally, there was no triangulation to support the experiences and perspectives of participants, which could have improved the accuracy of the information and provided a deeper, more nuanced understanding of the themes.

## Conclusions

This study demonstrates that both athletes and those who seek to support athletes understand that a diverse range of factors influence injury occurrence and recovery in Sri Lanka. These include the high demands of track and field, which often lead to physical overload and psychological stress. These problems are compounded by inadequate recovery, nutritional deficiencies, psychological stress, environmental hazards, limited access to appropriate equipment, and poor injury surveillance. Management practices varied widely and were influenced by financial constraints, lack of professional guidance, and reliance on informal or culturally driven approaches, affecting return-to-play decisions. Furthermore, athletes demonstrated limited engagement in self-monitoring practices and were often reliant on coaches rather than health-trained support, with cost frequently limiting access to physiotherapists and doctors. Stakeholders emphasized the need for improved injury surveillance systems and evidence-based approaches to injury prevention and management within a multidisciplinary team. These improvements have the potential to enhance recovery and injury prevention for track and field athletes in Sri Lanka.

## Supplementary Information

Supplementary Material 1.

Supplementary Material 2.

Supplementary Material 3.

Supplementary Material 4.

## Data Availability

The datasets used and/or analysed during the current study are available from the corresponding author on reasonable request.

## References

[1] O’Donovan G, Blazevich AJ, Boreham C, Cooper AR, Crank H, Ekelund U, et al. The ABC of physical activity for health: a consensus statement from the british association of sport and exercise sciences. J Sports Sci. 2010;28(6):573–91. 20401789 10.1080/02640411003671212

[2] Christakou A, Lavallee D. Rehabilitation from sports injuries: from theory to practice. Perspect Public Health. 2009;129(3):120–6. 19514635 10.1177/1466424008094802

[3] Jacobsson J, Timpka T, Kowalski J, Nilsson S, Ekberg J, Dahlström Ö, et al. Injury patterns in Swedish elite athletics: annual incidence, injury types and risk factors. Br J Sports Med. 2013;47(15):941–52. 23543425 10.1136/bjsports-2012-091651

[4] Hreljac A. Impact and overuse injuries in runners. Med Sci Sports Exerc. 2004;36(5):845–9. 15126720 10.1249/01.mss.0000126803.66636.dd

[5] Caine DJ, Harmer PA. Epidemiology of injury in olympic sports dennis J Caine epidemiology of injury in olympic sports et al international olympic committee and wiley-blackwell £108 518pp 9781405173643 1405173645 [Formula: see text]. Emerg Nurse. 2012;20(5):8. 10.7748/en.20.5.8.s2 27715201

[6] Lambert C, Reinert N, Stahl L, Pfeiffer T, Wolfarth B, Lachmann D, et al. Epidemiology of injuries in track and field athletes: a cross-sectional study of specific injuries based on time loss and reduction in sporting level. Phys Sportsmed. 2022;50(1):20–9. 33290132 10.1080/00913847.2020.1858701

[7] Edouard P, Navarro L, Branco P, Gremeaux V, Timpka T, Junge A. Injury frequency and characteristics (location, type, cause and severity) differed significantly among athletics (‘track and field’) disciplines during 14 international championships (2007–2018): implications for medical service planning. Br J Sports Med. 2020;54(3):159–67. 31722935 10.1136/bjsports-2019-100717

[8] Wolanin A, Hong E, Marks D, Panchoo K, Gross M. Prevalence of clinically elevated depressive symptoms in college athletes and differences by gender and sport. Br J Sports Med. 2016;50(3):167–71. 26782764 10.1136/bjsports-2015-095756

[9] Appaneal RN, Levine BR, Perna FM, Roh JL. Measuring postinjury depression among male and female competitive athletes. J Sport Exerc Psychol. 2009;31(1):60–76. 19325188 10.1123/jsep.31.1.60

[10] Walker D, Qureshi AW, Marchant D, Bahrami Balani A. Developing a simple risk metric for the effect of sport-related concussion and physical pain on mental health. PLoS ONE. 2023;18(10):e0292751. 37831707 10.1371/journal.pone.0292751 PMC10575528

[11] Feddermann-Demont N, Junge A, Edouard P, Branco P, Alonso JM. Injuries in 13 international Athletics championships between 2007–2012. Br J Sports Med. 2014;48(7):513–22. 24620039 10.1136/bjsports-2013-093087

[12] Ek A, Kowalski J, Jacobsson J. Training in spikes and number of training hours correlate to injury incidence in youth athletics (track and field): A prospective 52-week study. J Sci Med Sport. 2022;25(2):122–8. 34654650 10.1016/j.jsams.2021.09.006

[13] Timpka T, Jacobsson J, Dahlström Ö, Kowalski J, Bargoria V, Ekberg J, et al. The psychological factor ‘self-blame’ predicts overuse injury among top-level Swedish track and field athletes: a 12-month cohort study. Br J Sports Med. 2015;49(22):1472–7. 26373585 10.1136/bjsports-2015-094622

[14] Timpka T, Janson S, Jacobsson J, Dahlström Ö, Spreco A, Kowalski J, et al. Lifetime history of sexual and physical abuse among competitive athletics (track and field) athletes: cross sectional study of associations with sports and non-sports injury. Br J Sports Med. 2019;53(22):1412–7. 30190298 10.1136/bjsports-2018-099335

[15] Edouard P, Dandrieux P-E, Iatropoulos S, Blanco D, Branco P, Guex K. Injuries in athletics (track and field): A narrative review presenting the current problem of injuries. Z für Sportmedizin/German J sports Med. 2024;75(4):132–41.

[16] Timpka T, Fagher K, Bargoria V, Gauffin H, Andersson C, Jacobsson J, et al. ‘The little engine that could’: a qualitative study of medical service access and effectiveness among adolescent athletics athletes competing at the highest international level. Int J Environ Res Public Health. 2021;18(14):7278. 10.3390/ijerph18147278. 10.3390/ijerph18147278 PMC8304016 34299729

[17] Jacobsson J, Bergin D, Timpka T, Nyce JM, Dahlström Ö. Injuries in youth track and field are perceived to have multiple-level causes that call for ecological (holistic-developmental) interventions: A national sporting community perceptions and experiences. Scand J Med Sci Sports. 2018;28(1):348–55. 28605065 10.1111/sms.12929

[18] Walshe A, Daly E, Ryan L. A qualitative exploration of perceived challenges and opportunities in the implementation of injury prevention and management in amateur female sport. Front Sports Act Living. 2024;6:1430287. 39050790 10.3389/fspor.2024.1430287 PMC11266049

[19] Canata GL, D’Hooghe P, Hunt KJ, Kerkhoffs GM, Longo UG. Correction to: management of track and field injuries. Management of Track and Field Injuries. Cham: Springer International Publishing; 2022. p. C1-C1. 10.1007/978-3-030-60216-1.

[20] Wallace JB, Osmotherly PG, Gabbett TJ, Spratford W, Newman PM. Surveillance is the first step to preventing injury among fast jet aircrew: results of a 2-year prospective cohort study. Occup Environ Med. 2023;80(11):617–25. 37845016 10.1136/oemed-2023-108990

[21] McKay AK, Stellingwerff T, Smith ES, Martin DT, Mujika I, Goosey-Tolfrey VL, et al. Defining training and performance caliber: a participant classification framework. Int J Sports Physiol Perform. 2021;17(2):317–31. 10.1123/ijspp.2021-0451 34965513

[22] Braun V, Clarke V. Reflecting on reflexive thematic analysis. Qualitative Res Sport Exerc Health. 2019;11(4):589–97.

[23] Braun V, Clarke V. One size fits all? What counts as quality practice in (reflexive) thematic analysis? Qualitative Res Psychol. 2021;18(3):328–52.

[24] Braun V, Clarke V. Using thematic analysis in psychology. Qualitative Res Psychol. 2006;3(2):77–101.

[25] O’Brien BC, Harris IB, Beckman TJ, Reed DA, Cook DA. Standards for Reporting Qualitative Research: A Synthesis of Recommendations. Acad Med. 2014;89(9):1245–51. 24979285 10.1097/ACM.0000000000000388

[26] Tong A, Sainsbury P, Craig J. Consolidated criteria for reporting qualitative research (COREQ): a 32-item checklist for interviews and focus groups. Int J Qual Health Care. 2007;19(6):349–57. 17872937 10.1093/intqhc/mzm042

[27] Iatropoulos S, Edouard P. The ‘vicious circle’of sports injuries: an analysis of 165 athletics (track and field) athletes over a 39-week follow-up using Markov chains. BMJ Open Sport Exerc Med. 2025;11(2):e002420. 10.1136/bmjsem-2024-002420. 10.1136/bmjsem-2024-002420 PMC12182210 40546746

[28] Edouard P, Dandrieux PE, Tondut J, Chapon J, Navarro L, Ruffault A, et al. Injury risk reduction perceptions in athletics: survey on elite athletes and stakeholders participating at the Munich 2022 European Championships. German J Sports Medicine/Deutsche Z fur Sportmedizin. 2023;74(6). 10.5960/dzsm.2023.572.

[29] Prieto-González P, Martínez-Castillo JL, Fernández-Galván LM, Casado A, Soporki S, Sánchez-Infante J. epidemiology of sports-related injuries and associated risk factors in adolescent athletes: an injury surveillance. Int J Environ Res Public Health. 2021;18(9):4857. 10.3390/ijerph18094857. 10.3390/ijerph18094857 PMC8125505 34063226

[30] Pensgaard AM, Ivarsson A, Nilstad A, Solstad BE, Steffen K. Psychosocial stress factors, including the relationship with the coach, and their influence on acute and overuse injury risk in elite female football players. BMJ Open Sport Exerc Med. 2018;4(1):e000317. 29629182 10.1136/bmjsem-2017-000317 PMC5884339

[31] Tranaeus U, Martin S, Ivarsson A. Psychosocial risk factors for overuse injuries in competitive athletes: a mixed-studies systematic review. Sports Med. 2022;52(4):773–88. 34860356 10.1007/s40279-021-01597-5 PMC8938379

[32] Jayawardena R, Weerasinghe K, Madhujith T, Hills AP, Kalupahana N. Perceptions of the importance of sports nutrition knowledge and barriers in implementing them: a qualitative study among track and field stakeholders in Sri Lanka. BMC Nutr. 2024;10(1):17. 38263161 10.1186/s40795-023-00817-7 PMC10804860

[33] Cabre HE, Moore SR, Smith-Ryan AE, Hackney AC. Relative Energy Deficiency in Sport (RED-S): scientific, clinical, and practical implications for the female athlete. Dtsch Z Sportmed. 2022;73(7):225–34. 36479178 10.5960/dzsm.2022.546 PMC9724109

[34] Mason L, Connolly J, Devenney LE, Lacey K, O’Donovan J, Doherty R. Sleep, Nutrition, and injury risk in adolescent athletes: a narrative review. Nutrients. 2023;15(24). 10.3390/nu15245101. 10.3390/nu15245101 PMC10745648 38140360

[35] Li H, Moreland JJ, Peek-Asa C, Yang J. Preseason anxiety and depressive symptoms and prospective injury risk in collegiate athletes. Am J Sports Med. 2017;45(9):2148–55. 28441037 10.1177/0363546517702847

[36] Hausswirth C, Louis J, Aubry A, Bonnet G, Duffield R. Evidence of disturbed sleep and increased illness in overreached endurance athletes. Med Sci Sports Exerc. 2014;46(5):1036–45. 24091995 10.1249/MSS.0000000000000177

[37] Kerr ZY, Roos KG, Schmidt JD, Marshall SW. Prevention and management of physical and social environment risk factors for sports-related injuries. Am J Lifestyle Med. 2012;7(2):138–53.

[38] Segreti A, Celeski M, Guerra E, Crispino SP, Vespasiano F, Buzzelli L et al. Effects of environmental conditions on athlete’s cardiovascular system. J Clin Med. 2024;13(16):4961. 10.3390/jcm13164961. 10.3390/jcm13164961 PMC11355938 39201103

[39] Saragiotto BT, Di Pierro C, Lopes AD. Risk factors and injury prevention in elite athletes: a descriptive study of the opinions of physical therapists, doctors and trainers. Braz J Phys Ther. 2014;18(2):137–43. 24845023 10.1590/S1413-35552012005000147 PMC4183252

[40] Dudek S, Koziak W, Makieła M, Bętkowska A, Kornacka A, Dudek W, et al. Revolutionizing sports: the role of wearable technology and AI in training and performance analysis. Qual Sport. 2025;39:58456.

[41] Dijkstra HP, Pollock N, Chakraverty R, Alonso JM. Managing the health of the elite athlete: a new integrated performance health management and coaching model. Br J Sports Med. 2014;48(7):523–31. 24620040 10.1136/bjsports-2013-093222 PMC3963533

[42] Ekstrand J, Lundqvist D, Davison M, D’Hooghe M, Pensgaard AM. Communication quality between the medical team and the head coach/manager is associated with injury burden and player availability in elite football clubs. Br J Sports Med. 2019;53(5):304–8. 30104210 10.1136/bjsports-2018-099411 PMC6579487

[43] Ghrairi M, Loney T, Pruna R, Malliaropoulos N, Valle X. Effect of poor cooperation between coaching and medical staff on muscle re-injury in professional football over 15 seasons. Open Access J Sports Med. 2019;10:107–13. 31496844 10.2147/OAJSM.S221292 PMC6689085

[44] Kaur MN, Kumar S, Partap Y. Rehabilitation Strategies for sports injuries: a multidisciplinary perspective. Int J Sci Archit Technol Environ. 2025;2(5):234–42.

[45] Sprouse B, Chandran A, Rao N, Boltz AJ, Johnson M, Hennis P, et al. Injury and illness surveillance monitoring in team sports: a framework for all. Inj Epidemiol. 2024;11(1):23. 38858694 10.1186/s40621-024-00504-6 PMC11163858

[46] Ekegren CL, Donaldson A, Gabbe BJ, Finch CF. Implementing injury surveillance systems alongside injury prevention programs: evaluation of an online surveillance system in a community setting. Inj Epidemiol. 2014;1(1):19. 26613071 10.1186/s40621-014-0019-y PMC4648950

[47] Bae M. Biopsychosocial approach to sports injury: a systematic review and exploration of knowledge structure. BMC Sports Sci Med Rehabil. 2024;16(1):242. 39695836 10.1186/s13102-024-01025-x PMC11658233

