# Supplementary material for: “It’s never just one thing”: understanding risk factors for sports injuries in track and field
Source: BMC Sports Sci Med Rehabil. 2026 Apr 29;18:279. doi: 10.1186/s13102-026-01719-4 (PMC13274209; doi:10.1186/s13102-026-01719-4)
Supplement: Supplementary file 1 — Supplementary Material 1. [file 13102_2026_1719_MOESM1_ESM.docx]

**Supplementary Material 1. Guide for in-depth interviews with athletes and their parents**

**Introduction**

Thank you very much for taking the time to participate in this discussion. Today, we are interested in learning about your experiences and perceptions regarding the risk factors and possible causes of sports injuries in Sri Lankan track and field athletics. We want to hear your honest insights and reflections; there are no right or wrong answers. Your experiences are valuable, and your views will help us better understand how to prevent sports injuries in this field. As this is a qualitative study, the questions may vary slightly depending on how the conversation flows. You are encouraged to answer in a way that feels natural and comfortable. You are also free to skip any question that you do not wish to answer. If anything is unclear, feel free to ask for clarification at any time. With your permission, we would like to audio-record this session so that we can accurately capture your responses. Please be assured that all information you provide will be kept strictly confidential and used only for research purposes. Please feel relaxed, this is a conversation, not a test and we hope this session will be both meaningful and enjoyable for you.

**Details about the interview**

1. Date:
2. Time:
3. Name of the note taker:
4. Reference number of the athlete:
5. Details of the athletes/ parent (age, gender, event/ or child’s event):

*These questions are intended to guide discussion around broad topic areas; themes were not pre-defined and were developed inductively during data analysis.*

**Interview topic 1: Injury history and perceived causes**

**Main question: Can you share your experiences with past injuries (or your child’s injuries) and what you believe contributed to them?**

**Probes:**

1. Have you (or your child) ever experienced a sports injury? If so, what do you think was the immediate cause of the most recent injury?
2. What kind of impact did the injury have, such as missed competitions, missed training, financial burden, or emotional stress?
3. Can you describe the treatments or interventions you (or your child) used to recover from that injury?
4. How do you usually keep records of the injury severity and duration?

**Interview topic 2: Physical factors related to sports injuries**

**Main question: From your experience (or observation), what physical factors do you think lead to sports injuries or make them more likely to happen again?**

**Probes:**

1. After an injury, how do you (or your child) decide when to stop or continue training or competing?
2. In what way have past injuries made you (or your child) more likely to get injured again?
3. Which parts of physical training or preparation do you think work well, and which parts are missing or insufficient?
4. From your point of view, what types of muscle weaknesses or imbalances contributed to the injuries?

**Interview topic 3:** **Nutrition, recovery and psychological factors in injury risk**

**Main question: From your perspective, how do nutrition, recovery practices and mental health influence the risk of sports injuries?**

**Probes**

- 1. How do your eating and hydration habits (or those of your child) contribute to the risk of sports injuries?
  2. From your point of view and past experience, how might neglecting recovery increase the risk of injury?
  3. What recovery methods do you usually use (or observe your child using), such as sleep, massage, stretching, or ice baths, and how do they help prevent injuries?
  4. In what way has your mental health (or your child’s stress levels) influenced the risk of getting injured?

**Interview topic 4: Environmental and sports equipment-related risk factors**

**Main question: Can you describe how the training environment and equipment affect the risk of injury?**

**Probes**

1. Can you describe any injuries you (or your child) believe were caused by poor training surfaces, extreme weather conditions, or unsafe environments?
2. In your opinion, how suitable and safe are the training facilities you (or your child) regularly use, and what challenges have you faced in relation to these facilities that may affect injury risk?
3. In what ways do you think the type or condition of footwear (e.g., spikes, training shoes) or other training equipment may have contributed to an injury?
