# Supplementary material for: “It’s never just one thing”: understanding risk factors for sports injuries in track and field
Source: BMC Sports Sci Med Rehabil. 2026 Apr 29;18:279. doi: 10.1186/s13102-026-01719-4 (PMC13274209; doi:10.1186/s13102-026-01719-4)
Supplement: Supplementary file 2 — Supplementary Material 2. [file 13102_2026_1719_MOESM2_ESM.docx]

**Supplementary Material 2. Guide for in-depth interviews with athletic stakeholders**

**Introduction**

Thank you very much for taking the time to participate in this discussion. Today, we are interested in learning about your experiences and perceptions regarding the risk factors and possible causes of sports injuries in Sri Lankan track and field athletics. We want to hear your honest insights and reflections; there are no right or wrong answers. Your experiences are valuable, and your views will help us better understand how to prevent sports injuries in this field. As this is a qualitative study, the questions may vary slightly depending on how the conversation flows. You are encouraged to answer in a way that feels natural and comfortable. You are also free to skip any question that you do not wish to answer. If anything is unclear, feel free to ask for clarification at any time. With your permission, we would like to audio-record this session so that we can accurately capture your responses. Please be assured that all information you provide will be kept strictly confidential and used only for research purposes. Please feel relaxed, this is a conversation, not a test and we hope this session will be both meaningful and enjoyable for you.

**Details about the interview**

1. Date:
2. Time:
3. Name of the note taker:
4. Reference number of the athletic stakeholder:
5. Details of the athletic stakeholder
6. Age:
7. Gender:
8. Profession:
9. Number of years of experience:

*These questions are intended to guide discussion around broad topic areas; themes were not pre-defined and were developed inductively during data analysis.*

**Interview topic 1: Physical factors related to sports injuries**

**Main question: What physical factors do you believe contribute to the occurrence and recurrence of sports injuries in track and field?**

**Probes:**

1. In your opinion, when should an athlete stop training or competing after sustaining an injury?
2. How do you think previous injuries affect the risk of future injuries in athletes?
3. What aspects of an athlete’s physical preparation do you think are most effective or insufficient?
4. How do you believe weaknesses or asymmetries in certain muscle groups contribute to injuries?

**Interview topic 2:** **Nutrition, recovery and psychological factors in injury risk**

**Main question: From your perspective, how do nutrition, recovery practices and mental health influence an athlete’s risk of injury?**

**Probes**

- 1. How do you think athletes’ nutrition habits and hydration levels impact their risk of injury?
  2. In your view, how does neglecting recovery increase the likelihood of injury?
  3. What recovery strategies (e.g., sleep, massage, stretching, ice baths) do you think help reduce this risk?
  4. In what way do you think athletes’ mental health contributes to the risk of injury?

**Interview topic 3: Environmental and equipment-related risk factors**

**Main question: From your perspective, how do training environments, facilities, and equipment contribute to injury risks among athletes?**

**Probes**

1. How do you think poor training surfaces or extreme weather conditions influence injury risk in track and field athletes?
2. In your opinion, what challenges do athletes face in relation to the training facilities that may affect their risk of injury?
3. What role do you think footwear (e.g., spikes, training shoes) and other training equipment plays in either preventing or contributing to injuries?

**Interview topic 4: Injury monitoring and record-keeping practices**

**Main question: How do you currently monitor and document injuries among your athletes, and what improvements do you think are needed?**

**Probes**

1. Can you describe the method or system you currently use to keep track of your athletes' injuries?
2. What challenges or limitations have you experienced with this current method?
3. What changes or improvements would you suggest making injury tracking more accurate or effective?
4. In your view, why is it important to monitor the type, severity, and duration of injuries in athletes?
